# A New Transmuted Generalized Lomax Distribution: Properties and Applications to COVID-19 Data

**DOI:** 10.1155/2021/5918511

**Published:** 2021-10-07

**Authors:** Wael S. Abu El Azm, Ehab M. Almetwally, Sundus Naji AL-Aziz, Abd Al-Aziz H. El-Bagoury, Randa Alharbi, O. E. Abo-Kasem

**Affiliations:** ^1^Department of Statistics, Faculty of Commerce, Zagazig University, Zagazig, Egypt; ^2^Department of Statistics, Faculty of Business Administration, Delta University of Science and Technology, Mansoura 35511, Egypt; ^3^Department of Mathematical Sciences, College of Science, Princess Nourah bint Abdulrahman University, Riyadh, Saudi Arabia; ^4^Department of Mathematics, Faculty of Science, Tanta University, Tanta, Egypt; ^5^Department of Statistics, Faculty of Science, University of Tabuk, Tabuk, Saudi Arabia

## Abstract

A new five-parameter transmuted generalization of the Lomax distribution (TGL) is introduced in this study which is more flexible than current distributions and has become the latest distribution theory trend. Transmuted generalization of Lomax distribution is the name given to the new model. This model includes some previously unknown distributions. The proposed distribution's structural features, closed forms for an *r*th moment and incomplete moments, quantile, and Rényi entropy, among other things, are deduced. Maximum likelihood estimate based on complete and Type-II censored data is used to derive the new distribution's parameter estimators. The percentile bootstrap and bootstrap-t confidence intervals for unknown parameters are introduced. Monte Carlo simulation research is discussed in order to estimate the characteristics of the proposed distribution using point and interval estimation. Other competitive models are compared to a novel TGL. The utility of the new model is demonstrated using two COVID-19 real-world data sets from France and the United Kingdom.

## 1. Introduction

Many generators have been studied in recent years by expanding some effective classical distributions. Many applied fields, including dependability, demographics, engineering, economics, actuarial sciences, biological research, hydrology, insurance, medicine, and finance, have employed such created families of distributions for modeling and evaluating lifetime data. However, there are still a lot of real-world data occurrences that do not fit into any of the statistical distributions. Shaw and Buckley [1] introduced a new class of distributions known as transmuted distributions with cumulative distribution function (CDF) as

(1) $$\begin{array}{c} F_{T}\left(x\right)=\hbar \left(x\right)\left[1+\beta -\beta \;\hbar \left(x\right)\right]\text{;}\;\left|\beta \right|\leq 1\text{,} \end{array}$$

By differentiating equation (1), we get the probability density function (pdf) as follows:

(2) $$\begin{array}{c} f_{T}\left(x\right)=ℜ\left(x\right)\left[1+\beta -2\beta \;\hbar \left(x\right)\right], \end{array}$$

where *ℜ*(*x*) and  *ℏ*(*x*) are the base distribution's pdf and CDF.

There are various transmuted distributions suggested. Aryal and Tsokos [2] proposed the transmuted Weibull distribution as a new generalization of the Weibull distribution. Merovci [3] devised and explored the varied structural properties of the transmuted Rayleigh distribution. Khan and King [4] obtained the transmuted modified Weibull distribution. The transmuted Lomax distribution was presented by Ashour and Eltehiwy [5]. Transmuted Pareto distribution is introduced by Merovci and Puka [6]. The transmuted generalized linear exponential distribution was introduced by Elbatal et al. [7]; among others. Poboková and Michalková [8] proposed a transmuted Weibull distribution. Ali and Athar [9] have created a new generalized transmuted family of distributions (TD). They utilized Weibull distribution to generalized transmuted families of distributions (TWDn).

The Lomax distribution is a heavy-tail pdf popular in business, economics, and actuarial modeling. In some cases, it is also known as the Pareto Type-II distribution. In the event of a business failure, Lomax used it to fit data. It is essentially a Pareto distribution with a 0-support level. The pdf is as follows:

(3) $$\begin{array}{c} ℜ\left(x;\gamma ;\delta \right)=\gamma \;\delta {\left(1+\gamma x\right)}^{-\left(\delta +1\right)}\text{,}\;x\geq 0,\delta \text{ and }\gamma \;>\text{ 0.} \end{array}$$

The CDF for (3) is

(4) $$\begin{array}{c} \hbar \left(x;\gamma ;\delta \right)=\left[1-{\left(1+\gamma x\right)}^{-\delta }\right]\text{,}\;x\geq 0,\delta \text{ and }\gamma \;>\text{ 0,} \end{array}$$

where *δ* and *γ* are the shape and scale parameters, respectively.

The CDF and pdf of Gompertz-generalized G-family of distribution are given by Alizadeh et al. [10] as

(5) $$\begin{array}{c} F\left(x;\vartheta ;\pi \right)=1-e^{\left(\vartheta /\pi \right)\left\{1-{\left[1-\hbar \left(x\right)\right]}^{-\pi }\right\}};\;x\geq \text{0,}\pi \text{ and }\vartheta \;>\text{0,} \end{array}$$

(6) $$\begin{array}{c} f\left(x;\vartheta ;\pi \right)=\vartheta \;ℜ\left(x\right){\left[1-\hbar \left(x\right)\right]}^{-\pi -1}e^{\left(\vartheta /\pi \right)\left\{1-{\left[1-\hbar \left(x\right)\right]}^{-\pi }\right\}};\;x\geq 0,\pi \text{ and }\vartheta >0, \end{array}$$

where *ϑ* and *π* are extra form parameters that change the tail weights. *ℜ*(*x*) and  *ℏ*(*x*) are the parent (or baseline) distribution's pdf and CDF, respectively.

Now, if the density from (3) and (4) is replaced into (5) and (6), Oguntunde et al. [11] introduced a novel generalization of the Lomax distribution known as the Gompertz Lomax distribution (GoLom) with vector parameters *ƛ* where *ƛ*=(*δ*, *γ*, *ϑ*, *π*); the CDF and pdf are

(7) $$\begin{array}{c} F\left(x;ƛ\right)=\hbar \left(x;ƛ\right)=1-e^{\left(\vartheta /\pi \right)\left\{1-{\left[1+\gamma \;x\right]}^{\delta \;\pi }\right\}};\;x\geq \text{0,}\pi ,\delta ,\gamma \text{ and }\vartheta >\text{0.} \end{array}$$

The corresponding pdf is created by inserting the densities from (3) and (4) into (6) in the following order:

(8) $$\begin{array}{c} f\left(x;ƛ\right)=ℜ\left(x;ƛ\right)=\vartheta \delta \gamma {\left(1+\gamma x\right)}^{\delta \;\pi -1}e^{\left(\vartheta /\pi \right)\left\{1-{\left[1+\gamma x\right]}^{\delta \;\pi }\right\}};\;x\geq \text{0,}\pi ,\delta ,\gamma \text{ and }\vartheta >\text{ 0.} \end{array}$$

The transmuted generalized Lomax (TGL) distribution is a new five-parameter transmuted generalization of the Lomax distribution presented in this article. This generalization stems from a fundamental motivation as follows:

- Providing a very flexible life distribution that includes several new existing distributions as submodels
- Making a significant difference in data modeling

One of the advantages of this distribution is that it works on modeling COVID-19 data. In COVID-19, a new coronavirus disease has expanded worldwide since December 2019, producing over 218 million cases and over 4.5 million deaths, reported by the World Health Organization (WHO). There have been about 6.5 million cases of COVID-19 in France as of September 2021, with over 112850 deaths. There have been about 6.8 million cases of COVID-19 in the United Kingdom as of September 2021, with over 132740 deaths. Therefore, we decided to find the best mathematical-statistical model for modeling the data of the countries of France and the United Kingdom. There were also many researchers who worked on finding a model for these data, such as Almetwally [12], Almetwally [13], Almetwally [14], and others.

The following is a representation of how this article is structured. In Section 2, we define the new distribution. The new distribution's structural features are discussed in Section 3. The maximum likelihood estimators (MLEs) of parameters under complete and Type-II censored samples are investigated in Section 4.  Section 5 describes the various bootstrap confidence intervals.  Section 6 describes a Monte-Carlo simulation analysis using entire sample sizes and Type-II censored samples to estimate point and interval estimation of TGL distribution parameters. In Section 7, two real-world data sets are introduced, and at the end of the article, there is a conclusion.

## 2. Transmuted Generalized Lomax Model

The TGL distribution and its submodels are shown here. The CDF of the TGL distribution with vector parameters *ℤ* where *ℤ*=(*δ*, *γ*, *ϑ*, *π*, *β*) can be derived by substituting (7) and (8) in (1) and (2) as

(9) $$\begin{array}{c} F\left(x;\mathbb{Z}\right)=\left[1-e^{\vartheta /\pi \left[1-{\left(1+\gamma x\right)}^{\delta \;\pi }\right]}\right]\left[1+\beta e^{\vartheta /\pi \left[1-{\left(1+\gamma x\right)}^{\delta \pi }\right]}\right], \end{array}$$

and its pdf is as follows:

(10) $$\begin{array}{c} f\left(x;\mathbb{Z}\right)=\delta \vartheta \gamma {\left(1+\gamma x\right)}^{\delta \pi -1}e^{\vartheta /\pi \left[1-{\left(1+\gamma x\right)}^{\delta \pi }\right]}\left[1-\beta +2\beta e^{\vartheta /\pi \left[1-{\left(1+\gamma x\right)}^{\delta \pi }\right]}\right];\\ x>0,\;\pi ,\beta ,\delta ,\gamma \text{ and }\vartheta >\text{ 0.} \end{array}$$

As a result, the pdf (10) is defined as *X* ∼ TGL (*δ*, *γ*, *ϑ*, *π*, *β*).  Table 1 lists the TGL distribution's special submodels.

The survival (reliability) function $\bar{F}\left(x;\mathbb{Z}\right)$ and the hazard rate function *h*(*x*; *ℤ*) have the following definitions:

(11) $$\begin{array}{c} \bar{F}\left(x;\mathbb{Z}\right)=e^{\vartheta /\pi \left[1-{\left(1+\gamma x\right)}^{\delta \pi }\right]}\left\{1+\beta e^{\vartheta /\pi \left[1-{\left(1+\gamma x\right)}^{\delta \pi }\right]}\right\},\\ h\left(x;\mathbb{Z}\right)=\frac{\delta \vartheta \gamma {\left(1+\gamma x\right)}^{\delta \pi -1}\left[1-\beta +2\beta e^{\vartheta /\pi \left[1-{\left(1+\gamma x\right)}^{\delta \pi }\right]}\right]}{\left(1-\beta +\beta e^{\vartheta /\pi \left[1-{\left(1+\gamma x\right)}^{\delta \pi }\right]}\right)}. \end{array}$$

**Figure 1:**
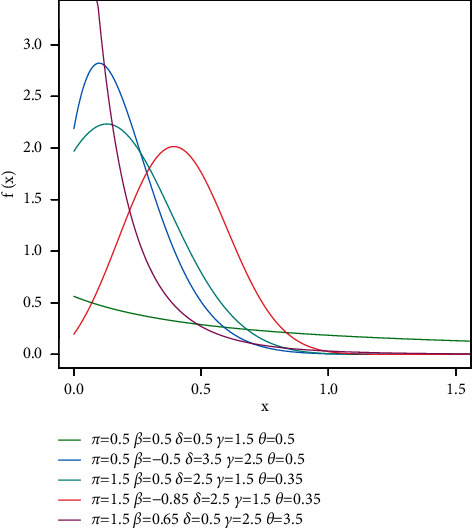
Plots of the TGL distribution's pdf.

For specific parameters selections, the pdf of TGL model is shown in Figure 1.

We can deduct from Figure 1 plots of the TGL distribution's pdf can be unimodal, normal, or right-skewed.

**Figure 2:**
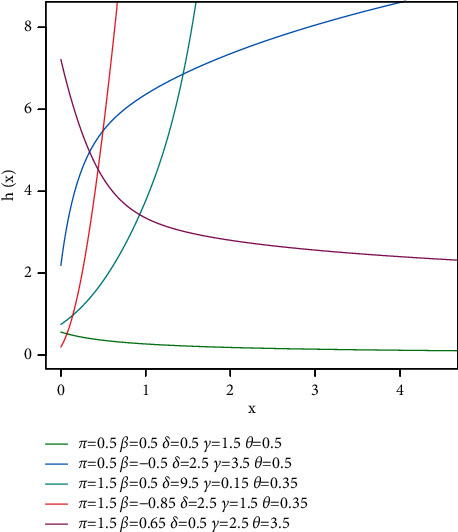
Plots showing the TGL distribution's hazard function.

We may derive from Figure 2 that the TGL distribution's hazard function can take the form of a decreasing, increasing, or upside-down shape.

**Lemma 1 .**

The TGL density function's limit is provided as

(12) $$\begin{array}{c} \underset{x⟶0}{\lim}f\left(x;\mathbb{Z}\right)=0,\\ \underset{x⟶\infty }{\lim}f\left(x;\mathbb{Z}\right)=0. \end{array}$$

**Proof**

The density function's conclusion is easy to illustrate (10).

Furthermore, the TGL hazard function's limit as *x*⟶0 is 0 and *x*⟶ ∞ is ∞ as shown as follows:

(13) $$\begin{array}{c} \underset{x⟶0}{\lim}h\left(x;\mathbb{Z}\right)=0,\\ \underset{x⟶\infty }{\lim}h\left(x;\mathbb{Z}\right)=\infty . \end{array}$$

This statement is simple to demonstrate.

## 3. Statistical Properties

The statistical aspects of the TGL distribution are examined in the following subsections, including moments, mode, quantile function, Rényi entropy, and order statistics.

### 3.1. Moments

The *r*^th^ instant near 0 is calculated. We can write as follows from (10).

(14) $$\begin{array}{c} \mu_{r}^{\prime }=\delta \vartheta \gamma \int_{0}^{\infty }x^{r}{\left(1+\gamma x\right)}^{\delta \pi -1}e^{{}^{\vartheta /\pi \left[1-{\left(1+\gamma x\right)}^{\delta \pi }\right]}}\left[1-\beta +2\beta e^{\vartheta /\pi \left[1-{\left(1+\gamma x\right)}^{\delta \pi }\right]}\right]\text{d}x=I_{1}+I_{2}. \end{array}$$

First, to obtain *I*_1_, as a result, we use binomial expansion.

(15) $$\begin{array}{c} I_{1}=\gamma^{-r}\left(1-\beta \right)\int_{0}^{\infty }{\left[{\left(\frac{\pi }{\vartheta }y+1\right)}^{1/\delta \;\pi }-1\right]}^{r}e^{-y}\text{d}y. \end{array}$$

So, *I*_1_ is given by

(16) $$\begin{array}{c} I_{1}=\gamma^{-r}\left(1-\beta \right)\sum_{i=0}^{r}\sum_{j=0}^{\infty }\left(\begin{array}{c} r\\ i \end{array}\right){\left(-1\right)}^{i}{\left(\frac{\pi }{\vartheta }\right)}^{\left(1/\delta \pi \right)\left(r-i\right)-j}\Gamma \left(\frac{1}{\delta \pi }\left(r-i\right)-j+1\right). \end{array}$$

In a similar way, I_2_ is as follows:

(17) $$\begin{array}{c} I_{2}=\beta \gamma^{-r}\sum_{i=0}^{r}\sum_{j=0}^{\infty }\left(\begin{array}{c} r\\ i \end{array}\right)\left(\begin{array}{c} \frac{1}{\delta \pi }\left(r-i\right)\\ \;j \end{array}\right){\left(-1\right)}^{i}{\left(\frac{\pi }{2\vartheta }\right)}^{\left(1/\delta \pi \right)\left(r-i\right)-j}\Gamma \left(\frac{1}{\delta \pi }\left(r-i\right)-j+1\right). \end{array}$$

Then, *μ*_*r*_′ can be expressed as

(18) $$\begin{array}{c} \mu_{r}^{\prime }=A\left(i,j\right)\gamma^{-r}\sum_{i=0}^{r}\sum_{j=0}^{\infty }\left(\begin{array}{c} r\\ i \end{array}\right)\left(\begin{array}{c} \frac{1}{\delta \pi }\left(r-i\right)\\ \;j \end{array}\right){\left(-1\right)}^{i}\Gamma \left(\frac{1}{\delta \pi }\left(r-i\right)-j+1\right)\\ \text{where}\\ A\left(i,j\right)=\left[\left(1-\beta \right){\left(\frac{\pi }{\vartheta }\right)}^{\left(1/\delta \pi \right)\left(r-i\right)-j}+\beta {\left(\frac{\pi }{2\vartheta }\right)}^{\left(1/\delta \pi \right)\left(r-i\right)-j}\right]. \end{array}$$

### 3.2. Mode

The mode of the TGL distribution was obtained in this subsection. The ln  *f*(*x*) is as follows:

ln  *f*(*x*)=ln(*δ* *γ* *ϑ*)+(*δπ* − 1)ln(1+*γx*)+*ϑ*/*π*[1 − (1+*γx*)^*δπ*^]+ln[1 − *β*+2*βe*^*ϑ*/*π*[1 − (1+*γx*)^*δπ*^]^]. The mode is the solution of the following equation:

(19) $$\begin{array}{c} 2\beta \left(\alpha \gamma -1\right)e^{\vartheta /\pi \left[1-{\left(1+\gamma x\right)}^{\delta \pi }\right]}+\left[\left(\delta \pi -1\right)-\delta \vartheta {\left(1+\gamma x\right)}^{\delta \pi }\right]\left(1-\beta \right)=0. \end{array}$$

### 3.3. Quantile and Median

By inverting cdf (9) as follows, the TGL distribution can be easily simulated: on (0, 1), if *U* follows a uniform distribution, then

(20) $$\begin{array}{c} q=\left(1-e^{\vartheta /\pi \left[1-{\left(1+\gamma x_{q}\right)}^{\delta \pi }\right]}\right)\left[1+\beta e^{\vartheta /\pi \left[1-{\left(1+\gamma x_{q}\right)}^{\delta \pi }\right]}\right]. \end{array}$$

Special Case *β*=1:

(21) $$\begin{array}{c} x_{0.5}=\frac{1}{\gamma }\left\{{\left[1+\frac{\pi }{2\vartheta }\ln\left(2\right)\right]}^{1/\delta \pi }-1\right\}. \end{array}$$

### 3.4. Rényi Entropy

The variance of the uncertainty is measured by the Rényi entropy of a pdf *f*(*x*) with random variable *X* of TGL distribution. The Rényi entropy is defined as for any real parameter *ψ* > 0  and *ψ* ≠ 1:

(22) $$\begin{array}{c} I_{R}\left(\psi \right)=\frac{1}{\psi -1}\log\int_{R}f^{\psi }\left(x\right)\text{d}x,\;\psi >0\text{ and }\psi \neq 1. \end{array}$$

We can extract the integrated component using the density function (10) as follows:

(23) $$\begin{array}{c} \int_{R}f^{\psi }\left(x\right)={\left(\delta \vartheta \gamma \right)}^{\psi }\int_{\text{0}}^{\infty }{\left[1+\gamma x\right]}^{\psi \left(\delta \;\pi -1\right)}e^{\left(\vartheta /\pi \right)\psi \left[1-{\left(1+\gamma x\right)}^{\delta \;\pi }\right]}{\left[1-\beta +2\beta e^{\left(\vartheta /\pi \right)\left[1-{\left(1+\gamma x\right)}^{\delta \pi }\right]}\right]}^{\psi }\text{d}x. \end{array}$$

The binomial expansion is then used as follows:

(24) $$\begin{array}{c} \int_{R}f^{\psi }\left(x\right)\text{d}x=\sum_{i,j=0}^{\infty }\sum_{l=0}^{j}\left(\begin{array}{c} \psi \\ i \end{array}\right)\left(\begin{array}{c} \psi \left(a-1\right)+bi\\ \;j \end{array}\right)\left(\begin{array}{c} j\\ l \end{array}\right){\left(-1\right)}^{i+j}\gamma^{l+2\psi }{\left[a\left(1+\beta \right)\right]}^{\psi -i}{\left[\beta \left(a+b\right)\right]}^{i}\\ \times \int_{0}^{\infty }x^{\psi +l}e^{-\gamma x\left(\psi +j\right)\text{d}x}, \end{array}$$

As a result, the TGL distribution's Rényi entropy is

(25) $$\begin{array}{c} I_{R}\left(\psi \right)={\frac{\left(\delta \vartheta \gamma \right)}{\psi }}^{\psi -1}\sum_{i=0}^{\infty }\sum_{j=0}^{\infty }\left(\begin{array}{c} \psi \\ i \end{array}\right)\left(\begin{array}{c} \left(1-\psi \right)\left(\frac{1}{\delta \pi }-1\right)\\ \;j \end{array}\right)\frac{{\left(1-\beta \right)}^{\psi -i}{\left(2\beta \right)}^{i}{\left(\pi /\psi \vartheta \right)}^{j}}{{\left(1+i\right)}^{j+1}}\Gamma \left(j+1\right). \end{array}$$

### 3.5. Order Statistics

In this part, we will look at single-order statistics for the TGL distribution. Let us say *x*_1_,…, *x*_*n*_; there are *n* TGL random variables that are both independent and identically distributed. Let *x*_(1)_, *x*_(2)_,…, *x*_(*n*)_ stand for the order statistics derived from these *n* variables. The pdf of the *r*^*th*^ order statistic, say *f*_*r*:*n*_(*x*), is then calculated as

(26) $$\begin{array}{c} f_{r:n}\left(x\right)=C_{r:n}{\left[F\left(x\right)\right]}^{r-1}f\left(x\right){\left[1-F\left(x\right)\right]}^{n-r}, \end{array}$$

where *C*_*r*:*n*_=*n*!/(*r* − 1)!(*n* − *r*)! The binomial expansion is used in this case; then, *r*^th^ order statistic of TGL distribution is

(27) $$\begin{array}{c} f_{r:n}\left(x\right)=\frac{n!}{\left(r-1\right)!\left(n-r\right)!}{\left[1-e^{\vartheta /\pi \left[1-{\left(1+\gamma x\right)}^{\delta \pi }\right]}\left(1+\beta e^{\vartheta /\pi \left[1-{\left(1+\gamma x\right)}^{\delta \pi }\right]}\right)\right]}^{r-1}\delta \vartheta \gamma {\left(1+\gamma x\right)}^{\delta \pi -1}e^{\vartheta /\pi \left[1-{\left(1+\gamma x\right)}^{\delta \pi }\right]}\\ \left[1-\beta +2\beta e^{\vartheta /\pi \left[1-{\left(1+\gamma x\right)}^{\delta \pi }\right]}\right]e^{\vartheta /\pi \left(n-r\right)\left[1-{\left(1+\gamma x\right)}^{\delta \pi }\right]}{\left(1-\beta +\beta e^{\vartheta /\pi \left[1-{\left(1+\gamma x\right)}^{\delta \pi }\right]}\right)}^{n-r}. \end{array}$$

For the TGL distribution, the *k*^th^ moments of *r*^th^ order statistics are

(28) $$\begin{array}{c} \mu_{r:n}^{\left(k\right)}=\frac{\delta \;\vartheta \;\gamma \;n!}{\left(r-1\right)!\left(n-r\right)!}\sum_{i=0}^{r-1}\sum_{j=0}^{n-r}\sum_{l=0}^{i}\left(\begin{array}{c} r-1\\ i \end{array}\right)\left(\begin{array}{c} n-r\\ j \end{array}\right)\left(\begin{array}{c} i\\ l \end{array}\right){\left(-1\right)}^{i}{\left(1-\beta \right)}^{n-r-j}\beta^{j+l}\\ \gamma^{-k}\int_{0}^{\infty }{\left\{{\left[1+\frac{\pi }{\vartheta }y\right]}^{1/\delta \pi }-1\right\}}^{k}e^{-\left(n-r+i+j+l+1\right)y}{\left[1+\frac{\pi }{\vartheta }y\right]}^{1-\left(1/\delta \pi \right)}\left[1-\beta +2\beta e^{-y}\right]\frac{1}{\delta \;\vartheta \;\gamma }{\left[1+\frac{\pi }{\vartheta }y\right]}^{\left(1/\delta \pi \right)-1}\text{d}y. \end{array}$$

By using the binomial expansion,

(29) $$\begin{array}{c} \mu_{r:n}^{\left(k\right)}=\frac{n!\gamma^{-k}}{\left(r-1\right)!\left(n-r\right)!}\sum_{i=0}^{r-1}\sum_{j=0}^{n-r}\sum_{l=0}^{i}\sum_{m=0}^{k}\sum_{t=0}^{\infty }\left(\begin{array}{c} r-1\\ i \end{array}\right)\left(\begin{array}{c} n-r\\ j \end{array}\right)\left(\begin{array}{c} i\\ l \end{array}\right)\left(\begin{array}{c} k\\ m \end{array}\right)\left(\begin{array}{c} k-m\\ \delta \pi \\ t \end{array}\right){\left(-1\right)}^{i+m}{\left(\frac{\pi }{\vartheta }\right)}^{t}\;\\ \times {\left(1-\beta \right)}^{n-r-j}\beta^{j+l}\int_{0}^{\infty }y^{t}e^{-\left(n-r+i+j+l+1\right)y}\left[1-\beta +2\beta e^{-y}\right]\text{d}y. \end{array}$$

For the TGL distribution, the *k*^th^ moments of *r*^th^ order statistics are

(30) $$\begin{array}{c} \mu_{r:n}^{\left(k\right)}=\frac{n!\gamma^{-k}}{\left(r-1\right)!\left(n-r\right)!}\sum_{i=0}^{r-1}\sum_{j=0}^{n-r}\sum_{l=0}^{i}\sum_{m=0}^{k}\sum_{t=0}^{\infty }\left(\begin{array}{c} r-1\\ i \end{array}\right)\left(\begin{array}{c} n-r\\ j \end{array}\right)\left(\begin{array}{c} i\\ l \end{array}\right)\left(\begin{array}{c} k\\ m \end{array}\right)\left(\begin{array}{c} k-m\\ \delta \pi \\ t \end{array}\right){\left(-1\right)}^{i+m}{\left(\frac{\pi }{\vartheta }\right)}^{t}{\left(1-\beta \right)}^{n-r-j}\beta^{j+l}\\ \times \left[\frac{1-\beta }{{\left(n-r+i+j+l+1\right)}^{t+1}}+\frac{2\beta }{{\left(n-r+i+j+l+2\right)}^{t+1}}\right]\Gamma \left(t+1\right). \end{array}$$

## 4. Parameter Estimation

The MLEs of the parameters *ℤ*=(*δ*, *γ*, *ϑ*, *π*, *β*) under complete and Type-II censored samples are investigated in this section. Approximate confidence intervals (ACIs) for unknown values are also calculated using the Fisher information matrix.

### 4.1. MLEs under Complete Sample

In the statistical literature, various approaches for parameter estimation have been given, with the MLEs method being the most extensively employed. We explore applying MLEs to estimate the parameters of the TGL distribution with a complete sample. If *x*_1_, *x*_2_,…, *x*_*n*_ is a random sample of this distribution of size *n* with a set of parameter vectors *ℤ*=(*δ*, *γ*, *ϑ*, *π*, *β*), then the log-likelihood function, say *ℓ*_1_(*ℤ*), may be stated as

(31) $$\begin{array}{c} \ell_{1}\left(\mathbb{Z}\right)=n\left[\ln\left(\vartheta \right)+\ln\left(\delta \right)+\ln\left(\gamma \right)\right]+\left(\delta \;\pi -1\right)\sum_{i=1}^{n}\ln\left(1+\gamma x_{i}\right)+\frac{\vartheta }{\pi }\sum_{i=1}^{n}\psi_{i}+\sum_{i=1}^{n}\ln\left[1-\beta +2\beta e^{\left(\vartheta /\pi \right)\psi_{i}}\right], \end{array}$$

where *ψ*_*i*_=[1 − (1+*γx*_*i*_)^*δ* *π*^]. The partial differential equations *ℓ*_1_(*ℤ*) are calculated as follows:

(32) $$\begin{array}{c} \frac{\partial \ell_{1}\left(\mathbb{Z}\right)}{\partial \delta }=\frac{n}{\delta }+\pi \sum_{i=1}^{n}\ln\left(1+\gamma x_{i}\right)-\vartheta \sum_{i=1}^{n}{\left(1+\gamma x_{i}\right)}^{\delta \pi }\ln\left(1+\gamma x_{i}\right)-\sum_{i=1}^{n}\frac{2\beta \vartheta {\left(1+\gamma x_{i}\right)}^{\delta \pi }\ln\left(1+\gamma x_{i}\right)e^{\left(\vartheta /\pi \right)\psi_{i}}}{1-\beta +2\beta e^{\left(\vartheta /\pi \right)\psi_{i}}},\\ \frac{\partial \ell_{1}\left(\mathbb{Z}\right)}{\partial \vartheta }=\frac{n}{\vartheta }+\frac{1}{\pi }\sum_{i=1}^{n}\psi_{i}+\sum_{i=1}^{k}\frac{\psi_{i}/\pi e^{\left(\vartheta /\pi \right)\psi_{i}}}{1-\beta +2\beta e^{\left(\vartheta /\pi \right)\psi_{i}}},\\ \frac{\partial \ell_{1}\left(\mathbb{Z}\right)}{\partial \gamma }=\frac{n}{\gamma }+\left(\delta \;\pi -1\right)\sum_{i=1}^{n}\frac{x_{i}}{\left(1+\gamma x_{i}\right)}-\delta \vartheta \sum_{i=1}^{n}x_{i}{\left(1+\gamma x_{i}\right)}^{\delta \pi -1}-\sum_{i=1}^{n}\frac{2\beta \delta \vartheta e^{\left(\vartheta /\pi \right)\psi_{i}}\left(x_{i}{\left(1+\gamma x_{i}\right)}^{\delta \pi -1}\right)}{1-\beta +2\beta e^{\left(\vartheta /\pi \right)\psi_{i}}},\\ \frac{\partial \ell_{1}\left(\mathbb{Z}\right)}{\partial \pi }=\delta \sum_{i=1}^{n}\ln\left(1+\gamma x_{i}\right)-\frac{\vartheta }{\pi^{2}}\sum_{i=1}^{n}\pi \delta {\left(1+\gamma x_{i}\right)}^{\delta \pi }\ln\left(1+\gamma x_{i}\right)+\psi_{i}\\ -\sum_{i=1}^{n}\frac{2\beta e^{\left(\vartheta /\pi \right)\psi_{i}}\left(\vartheta \delta \pi^{-1}{\left(1+\gamma x_{i}\right)}^{\delta \pi }\ln\left(1+\gamma x_{i}\right)-\psi_{i}\right)}{1-\beta +2\beta e^{\left(\vartheta /\pi \right)\psi_{i}}},\\ \frac{\partial \ell_{1}\left(\mathbb{Z}\right)}{\partial \beta }=\sum_{i=1}^{n}\frac{2e^{\left(\vartheta /\pi \right)\psi_{i}}-1}{1-\beta +2\beta e^{\left(\vartheta /\pi \right)\psi_{i}}}. \end{array}$$

The nonlinear equations are numerically solved to determine ML estimators as ∂*ℓ*_1_(*ℤ*)/∂*δ*=0, ∂*ℓ*_1_(*ℤ*)/∂*γ*=0, ∂*ℓ*_1_(*ℤ*)/∂*ϑ*=0, and ∂*ℓ*_1_(*ℤ*)/∂*π*=0∂*ℓ*_1_(*ℤ*)/∂*β*=0 using an iterative technique.

### 4.2. MLEs under Type-II Censored Sample

The MLEs of parameters for TGL distribution based on Type-II censored samples are investigated in this subsection. The Fisher information matrix for Type-II censored model is also used to calculate the approximate confidence intervals for the unknown parameters *ℤ*=(*δ*, *γ*, *ϑ*, *π*, *β*). Let *x*_1_, *x*_2_,…, *x*_*n*_ be a random sample of size *n*, and we only look at the first k-th order statistics based on the Type-II censored sample. Likelihood function in this scenario is of the kind

(33) $$\begin{array}{c} L_{2}\left(\mathbb{Z}\right)=C\prod_{i=1}^{k}f\left(x_{i:k:n}\right){\left[1-F\left(x_{i:k:n}\right)\right]}^{n-k},\;x_{1:k:n}\leq x_{2:k:n}\leq \cdots \leq x_{k:k:n}. \end{array}$$

where *C* is a constant and *x*_1:*k*:*n*_, *x*_2:*k*:*n*_,…, *x*_*k*:*k*:*n*_ is the data that has been censored. The log-likelihood function *ℓ*_2_(*ℤ*) is possibly written as follows without constant term from (15).

(34) $$\begin{array}{c} \ell_{2}\left(\mathbb{Z}\right)=k\left[\ln\left(\vartheta \right)+\ln\left(\delta \right)+\ln\left(\gamma \right)\right]+\left(\delta \;\pi -1\right)\sum_{i=1}^{k}\ln\left(1+\gamma x_{i}\right)+\frac{\vartheta }{\pi }\sum_{i=1}^{k}\psi_{i}+\sum_{i=1}^{k}\ln\left[1-\beta +2\beta e^{\left(\vartheta /\pi \right)\psi_{i}}\right]\;\\ +\left(n-k\right)\sum_{i=1}^{k}\ln\left[1-\left[1-e^{\left(\vartheta /\pi \right)\psi_{i}}\right]\left[1+\beta e^{\left(\vartheta /\pi \right)\psi_{i}}\right]\right], \end{array}$$

where *x*_*i*_=*x*_*i*:*k*:*n*_, *i*=1,2,…, *k*, denotes the time of the k-th failure and *x*_*k*_ denotes the time of the k-th failure. The MLEs *ℤ*=(*δ*, *γ*, *ϑ*, *π*, *β*) are the solutions to the following five equations:

(35) $$\begin{array}{c} \frac{\partial \ell_{2}\left(\mathbb{Z}\right)}{\partial \vartheta }=\frac{k}{\vartheta }+\frac{1}{\pi }\sum_{i=1}^{k}\psi_{i}+\sum_{i=1}^{k}\frac{\left(\psi_{i}/\pi \right)e^{\left(\vartheta /\pi \right)\psi_{i}}}{1-\beta +2\beta e^{\left(\vartheta /\pi \right)\psi_{i}}}+\left(n-k\right)\sum_{i=1}^{k}\frac{-\left(\beta \psi_{i}/\pi \right)e^{\left(\vartheta /\pi \right)\psi_{i}}+\left(\psi_{i}/\pi \right)e^{\left(\vartheta /\pi \right)\psi_{i}}+\left(2\beta \psi_{i}/\pi \right)e^{\left(2\vartheta /\pi \right)\psi_{i}}}{1-\left[1-e^{\left(\vartheta /\pi \right)\psi_{i}}\right]\left[1+\beta e^{\left(\vartheta /\pi \right)\psi_{i}}\right]}=0,\\ \frac{\partial \ell_{2}\left(\mathbb{Z}\right)}{\partial \delta }=\frac{k}{\delta }+\pi \sum_{i=1}^{k}\ln\left(1+\gamma x_{i}\right)-\vartheta \sum_{i=1}^{k}{\left(1+\gamma x_{i}\right)}^{\delta \pi }\ln\left(1+\gamma x_{i}\right)-\sum_{i=1}^{k}\frac{2\beta \vartheta {\left(1+\gamma x_{i}\right)}^{\delta \pi }\ln\left(1+\gamma x_{i}\right)e^{\left(\vartheta /\pi \right)\psi_{i}}}{1-\beta +2\beta e^{\left(\vartheta /\pi \right)\psi_{i}}}\;\\ +\left(n-k\right)\sum_{i=1}^{k}\frac{\vartheta {\left(1+\gamma x_{i}\right)}^{\delta \pi }\ln\left(1+\gamma x_{i}\right)e^{\left(\vartheta /\pi \right)\psi_{i}}\left[\beta -1-2\beta e^{\left(\vartheta /\pi \right)\psi_{i}}\right]}{1-\left[1-e^{\left(\vartheta /\pi \right)\psi_{i}}\right]\left[1+\beta e^{\left(\vartheta /\pi \right)\psi_{i}}\right]}=0, \end{array}$$

(36) $$\begin{array}{c} \frac{\partial \ell_{2}\left(\mathbb{Z}\right)}{\partial \pi }=\delta \sum_{i=1}^{k}\ln\left(1+\gamma x_{i}\right)-\frac{\vartheta }{\pi^{2}}\sum_{i=1}^{k}\pi \delta {\left(1+\gamma x_{i}\right)}^{\delta \pi }\ln\left(1+\gamma x_{i}\right)+\psi_{i}\\ -\sum_{i=1}^{k}\frac{2\beta e^{\left(\vartheta /\pi \right)\psi_{i}}\left(\vartheta \delta \pi^{-1}{\left(1+\gamma x_{i}\right)}^{\delta \pi }\ln\left(1+\gamma x_{i}\right)-\psi_{i}\right)\;}{1-\beta +2\beta e^{\left(\vartheta /\pi \right)\psi_{i}}}\;\\ -\left(n-k\right)\sum_{i=1}^{k}\frac{\left(\delta \vartheta \pi^{-1}{\left(1+\gamma x_{i}\right)}^{\delta \pi }\ln\left(1+\gamma x_{i}\right)e^{\left(\vartheta /\pi \right)\psi_{i}}\left(\beta -1-2\beta e^{\left(\vartheta /\pi \right)\psi_{i}}\right)-\psi_{i}\right)}{1-\left[1-e^{\left(\vartheta /\pi \right)\psi_{i}}\right]\left[1+\beta e^{\left(\vartheta /\pi \right)\psi_{i}}\right]}=0,\\ -\left(n-k\right)\sum_{i=1}^{k}\frac{\delta \vartheta e^{\left(\vartheta /\pi \right)\psi_{i}}\left(x_{i}{\left(1+\gamma x_{i}\right)}^{\delta \pi -1}\right)\left(\beta -1-\beta e^{\left(\vartheta /\pi \right)\psi_{i}}\right)}{1-\left[1-e^{\left(\vartheta /\pi \right)\psi_{i}}\right]\left[1+\beta e^{\left(\vartheta /\pi \right)\psi_{i}}\right]}=0,\\ \frac{\partial \ell_{2}\left(\mathbb{Z}\right)}{\partial \beta }=\sum_{i=1}^{k}\frac{2e^{\left(\vartheta /\pi \right)\psi_{i}}-1}{1-\beta +2\beta e^{\left(\vartheta /\pi \right)\psi_{i}}}+\left(n-k\right)\sum_{i=1}^{k}\frac{e^{\left(\vartheta /\pi \right)\psi_{i}}\left(e^{\left(\vartheta /\pi \right)\psi_{i}}-1\right)}{1-\left[1-e^{\left(\vartheta /\pi \right)\psi_{i}}\right]\left[1+\beta e^{\left(\vartheta /\pi \right)\psi_{i}}\right]}. \end{array}$$

(37) $$\begin{array}{c} \frac{\partial \ell_{2}\left(\mathbb{Z}\right)}{\partial \gamma }=\frac{k}{\gamma }+\left(\delta \pi -1\right)\sum_{i=1}^{k}\frac{x_{i}}{\left(1+\gamma x_{i}\right)}-\delta \vartheta \sum_{i=1}^{k}x_{i}{\left(1+\gamma x_{i}\right)}^{\delta \pi -1}-\sum_{i=1}^{k}\frac{2\beta \delta \vartheta e^{\left(\vartheta /\pi \right)\psi_{i}}\left(x_{i}{\left(1+\gamma x_{i}\right)}^{\delta \pi -1}\right)}{1-\beta +2\beta e^{\left(\vartheta /\pi \right)\psi_{i}}}\\ -\left(n-k\right)\sum_{i=1}^{k}\frac{\delta \vartheta e^{\left(\vartheta /\pi \right)\psi_{i}}\left(x_{i}{\left(1+\gamma x_{i}\right)}^{\delta \pi -1}\right)\left(\beta -1-\beta e^{\left(\vartheta /\pi \right)\psi_{i}}\right)}{1-\left[1-e^{\left(\vartheta /\pi \right)\psi_{i}}\right]\left[1+\beta e^{\left(\vartheta /\pi \right)\psi_{i}}\right]}=0,\\ \frac{\partial \ell_{2}\left(\mathbb{Z}\right)}{\partial \beta }=\sum_{i=1}^{k}\frac{2e^{\left(\vartheta /\pi \right)\psi_{i}}-1}{1-\beta +2\beta e^{\left(\vartheta /\pi \right)\psi_{i}}}+\left(n-k\right)\sum_{i=1}^{k}\frac{e^{\left(\vartheta /\pi \right)\psi_{i}}\left(e^{\left(\vartheta /\pi \right)\psi_{i}}-1\right)}{1-\left[1-e^{\left(\vartheta /\pi \right)\psi_{i}}\right]\left[1+\beta e^{\left(\vartheta /\pi \right)\psi_{i}}\right]}. \end{array}$$

It is to be noted that equation (16) cannot be solved explicitly. To obtain the MLEs *ℤ*=(*δ*, *γ*, *ϑ*, *π*, *β*), a numerical approach is required, and a numerical technique is needed. We obtain the observed Fisher information matrix since its expectation requires numerical integration. The 5  ×  5 observed information or Hessian matrix *H*(*ℤ*) is

(38) $$\begin{array}{c} H\left(\mathbb{Z}\right)=\left[\begin{array}{ccccc} H_{\delta \delta } & H_{\delta \gamma } & H_{\delta \vartheta } & H_{\delta \pi } & H_{\delta \beta }\\ H_{\gamma \delta } & H_{\gamma \gamma } & H_{\gamma \vartheta } & H_{\gamma \pi } & H_{\gamma \beta }\\ H_{\vartheta \delta } & H_{\vartheta \gamma } & H_{\vartheta \vartheta } & H_{\vartheta \pi } & H_{\vartheta \beta }\\ H_{\pi \delta } & H_{\pi \gamma } & H_{\pi \vartheta } & H_{\pi \pi } & H_{\pi \beta }\\ H_{\beta \delta } & H_{\beta \gamma } & H_{\beta \vartheta } & H_{\beta \pi } & H_{\beta \beta } \end{array}\right]. \end{array}$$

The Fisher information matrix *H*(*ℤ*) is given by the negative expected of second partial derivatives of (15) for the unknown parameters *ℤ*=(*δ*, *γ*, *ϑ*, *π*, *β*) locally at $\left(\hat{\delta },\hat{\gamma },\hat{\vartheta },\hat{\pi },\hat{\beta }\right)$ given in (16). Under some regularity conditions, $\left(\hat{\delta },\hat{\gamma },\hat{\vartheta },\hat{\pi },\hat{\beta }\right)$ is approximately normal with mean (*δ*, *γ*, *ϑ*, *π*, *β*) and covariance matrix *H*^−1^(*ℤ*). Practically, we estimate *H*^−1^(*ℤ*) by$H_{\left(\hat{\delta },\hat{\gamma },\hat{\vartheta },\hat{\pi },\hat{\beta }\right)}^{-1}$; then

(39) $$\begin{array}{c} I_{\left(\hat{\delta },\hat{\gamma },\hat{\vartheta },\hat{\pi },\hat{\beta }\right)}^{-1}=-{\left[\begin{array}{ccccc} H_{\delta \delta } & H_{\delta \gamma } & H_{\delta \vartheta } & H_{\delta \pi } & H_{\delta \beta }\\ H_{\gamma \delta } & H_{\gamma \gamma } & H_{\gamma \vartheta } & H_{\gamma \pi } & H_{\gamma \beta }\\ H_{\vartheta \delta } & H_{\vartheta \gamma } & H_{\vartheta \vartheta } & H_{\vartheta \pi } & H_{\vartheta \beta }\\ H_{\pi \delta } & H_{\pi \gamma } & H_{\pi \vartheta } & H_{\pi \pi } & H_{\pi \beta }\\ H_{\beta \delta } & H_{\beta \gamma } & H_{\beta \vartheta } & H_{\beta \pi } & H_{\beta \beta } \end{array}\right]}_{\left(\hat{\delta },\hat{\gamma },\hat{\vartheta },\hat{\pi },\hat{\beta }\right)}^{-1},\\ H_{\left(\hat{\delta },\hat{\gamma },\hat{\vartheta },\hat{\pi },\hat{\beta }\right)}^{-1}=\left[\begin{array}{ccccc} \text{Var}\left(\hat{\delta }\right) & \text{Cov}\left(\hat{\delta },\hat{\gamma }\right) & \text{Cov}\left(\hat{\delta },\hat{\vartheta }\right) & \text{Cov}\left(\hat{\delta },\hat{\pi }\right) & \text{Cov}\left(\hat{\delta },\hat{\beta }\right)\\ \text{Cov}\left(\hat{\gamma },\hat{\delta }\right) & \text{Var}\left(\hat{\gamma }\right) & \text{Cov}\left(\hat{\gamma },\hat{\vartheta }\right) & \text{Cov}\left(\hat{\gamma },\hat{\pi }\right) & \text{Cov}\left(\hat{\gamma },\hat{\beta }\right)\\ \text{Cov}\left(\hat{\vartheta },\hat{\delta }\right) & \text{Cov}\left(\hat{\vartheta },\hat{\gamma }\right) & \text{Var}\left(\hat{\vartheta }\right) & \text{Cov}\left(\hat{\vartheta },\hat{\pi }\right) & \text{Cov}\left(\hat{\vartheta },\hat{\beta }\right)\\ \text{Cov}\left(\hat{\pi },\hat{\delta }\right) & \text{Cov}\left(\hat{\pi },\hat{\gamma }\right) & \text{Cov}\left(\hat{\pi },\hat{\vartheta }\right) & \text{Var}\left(\hat{\pi }\right) & \text{Cov}\left(\hat{\pi },\hat{\beta }\right)\\ \text{Cov}\left(\hat{\beta },\hat{\delta }\right) & \text{Cov}\left(\hat{\beta },\hat{\gamma }\right) & \text{Cov}\left(\hat{\beta },\hat{\vartheta }\right) & \text{Cov}\left(\hat{\beta },\hat{\pi }\right) & \text{Var}\left(\hat{\beta }\right) \end{array}\right]. \end{array}$$

The elements of the observed Hessian matrix are computed using an iterative numerical solution method. Now, the ACIs *ℤ*=(*δ*, *γ*, *ϑ*, *π*, *β*) can be obtained as follows:

(40) $$\begin{array}{c} \left\{\begin{array}{c} \hat{\delta }\pm z_{1-\left(\alpha /2\right)}.\sqrt{\text{Var}\left(\hat{\delta }\right),}\\ \hat{\gamma }\pm z_{1-\left(\alpha /2\right)}.\sqrt{\text{Var}\left(\hat{\gamma }\right)},\\ \hat{\vartheta }\pm z_{1-\left(\alpha /2\right)}.\sqrt{\text{Var}\left(\hat{\vartheta }\right)},\\ \hat{\pi }\pm z_{1-\left(\alpha /2\right)}.\sqrt{\text{Var}\left(\hat{\pi }\right)},\\ \hat{\beta }\pm z_{1-\left(\alpha /2\right)}.\sqrt{\text{Var}\left(\hat{\beta }\right)}, \end{array}\right. \end{array}$$

where *z*_*α*_ is the 100 *α* − th percentile of a standard normal distribution.

## 5. Bootstrap Confidence Interval

We create two parametric bootstrap confidence intervals (CI) *ℤ*=(*δ*, *γ*, *ϑ*, *π*, *β*) in this section as follows.

### 5.1. Percentile Bootstrap (Boot-P)

1. Compute the MLE of *ℤ*=(*δ*, *γ*, *ϑ*, *π*, *β*) based on complete and censored samples
2. Generate a bootstrap sample using *ℤ* to obtain the bootstrap estimate of *ℤ* say ${\hat{\mathbb{Z}}}^{\text{b}}$ using the bootstrap sample
3. Repeat step (2) B times to get (*ℤ*^*b*(1)^, *ℤ*^*b*(2)^,…, *ℤ*^*b*(*B*)^).
4. Arrange (*ℤ*^*b*(1)^, *ℤ*^*b*(2)^,…, *ℤ*^*b*(*B*)^) in order of ascending as (*ℤ*^*b*[1]^, *ℤ*^*b*[2]^,…, *ℤ*^*b*[*B*]^).
5. A two-side 100(1 − *γ*)% Boot-P CI for the unknown parameters *ℤ*=(*δ*, *γ*, *ϑ*, *π*, *β*) is set by $\left({\hat{\mathbb{Z}}}^{b\left[B\left(\gamma /2\right)\right]},{\hat{\mathbb{Z}}}^{b\left[B\left(1-\gamma /2\right)\right]}\right)$

### 5.2. Bootstrap-t (Boot-t)

1. The same steps as (1-2) in Boot-p
2. Compute the t-statistic of $\mathbb{Z}=\left(\delta ,\gamma ,\vartheta ,\pi ,\beta \right)T=\left({\hat{\mathbb{Z}}}^{\text{b}}-\hat{\mathbb{Z}}\right)/\sqrt{Var\left({\hat{\mathbb{Z}}}^{\text{b}}\right)}$ where $Var\left({\hat{\mathbb{Z}}}^{\text{b}}\right)$ asymptotic variances of $\hat{\mathbb{Z}}=\left(\hat{\delta },\hat{\gamma },\hat{\vartheta },\hat{\pi },\hat{\beta }\right)$ and it can be obtained using the Fisher information matrix
3. Repeat steps 2-3 B times and obtain *T*^(1)^, *T*^(2)^,…, *T*^(*B*)^
4. Arrange *T*^(1)^, *T*^(2)^,…, *T*^(*B*)^ in ascending order as *T*^(1)^, *T*^(2)^,…, *T*^(*B*)^
5. A two-side 100(1 − *γ*)% Boot-t CI for the unknown parameters *ℤ*=(*δ*, *γ*, *ϑ*, *π*, *β*) is given by

(41) $$\begin{array}{c} \left\{\hat{\mathbb{Z}}+T^{\left[B\left(\gamma /2\right)\right]}\sqrt{\text{Var}\left(\hat{\mathbb{Z}}\right)},\hat{\mathbb{Z}}+T^{\left[B\left(1-\gamma /2\right)\right]}\sqrt{\text{Var}\left(\hat{\mathbb{Z}}\right)}\right\}. \end{array}$$

## 6. Simulation Study

In this section, we discuss the Monte Carlo simulation study to estimate point and interval estimation of parameters of TGL distribution based on complete sample sizes and Type-II censored samples. The simulation results are in Tables 2– 5, and concluding remarks of simulation results are obtained in this section. A Monte Carlo simulation is an initial task for studying different properties of parameters of the TGL model based on different sample schemes; we can use these steps:

1. Generate random sample from a uniform distribution with intervals 0 and 1 and sample size *n* as 30, 50, and 100
2. Determine different actuals values of parameters of TGL distribution as
3. Case 1: *π*=0.3,  *β*=0.3,  *δ*=0.5,  *γ*=0.6,  *ϑ*=0.5
4. Case 2: *π*=1.2,  *β*=0.4,  *δ*=0.8,  *γ*=0.75,  *ϑ*=1.9
5.  Case 3: *π*=0.35,  *β*=−0.4,  *δ*=0.45,  *γ*=0.75,  *ϑ*=0.85
6.  Case 4: *π*=1.5,  *β*=−0.6,  *δ*=1.4,  *γ*=2.7,  *ϑ*=1.8
7. We use the inverse CDF method to transform the CDF in terms of *u* and get the sample of TGL distribution
8. Sort sample and select the first *m* failures as 20 and 25 where *n* = 30, 30, and 40 where *n* = 50 and 70 and 90 where *n* = 100
9. By using different programs as Mathcad, R-software, Mable, and Mathematica, we can obtain the results of the simulation
10. We use 10000 iterations in the summation generator
11. In point estimation, we obtain bias and the mean squared error (MSE) of parameters of TGL distribution
12. In intervals estimation, we obtain a length of CI for ACI denoted as L.ACI, length of percentile bootstrap CI can be denoted as L.BPCI, length of bootstrap-t CI can be denoted as L.BTCI, and coverage probability (CP) of ACI

## 7. Concluding Remarks of Simulation Results

Tables 2–5 show the simulation results of point and interval estimates of TGL distribution parameters using Type-II censored samples and entire sample sizes. Based on these Tables, the following concluding remakes are noticed.

- As sample size increases and fixed other values of the model, the various measures for the parameter of TGL distribution estimates decrease
- As the number of units to be failed increases and fixed other values of the model, the various measures for the parameter of TGL distribution Type-II censored samples estimates decrease
- The bootstrap is the shortest length of CI for interval estimation of parameter TGL distribution

## 8. Real Data Analysis

The relevance and potentiality of the TGL distribution are demonstrated in this section through the application of two real data sets.

### 8.1. Data Set (1): COVID-19 of France

The COVID-19 data in question is from France, and it covers a period of 108 days, from March 1 to June 16, 2021. This data was formed by using daily new deaths (ND), daily cumulative cases (CC), and daily cumulative deaths (CD) as follows:

(42) $$\begin{array}{c} x_{i}=\frac{{\text{ND}}_{i}}{{\text{CC}}_{i}-{\text{CD}}_{i-1}}\times 1000. \end{array}$$

The data are as follows: 0.0024 0.0025 0.0027 0.0045 0.0045 0.0062 0.0109 0.0113 0.0123 0.0123 0.0126 0.0129 0.0130 0.0139 0.0152 0.0160 0.0160 0.0161 0.0164 0.0169 0.0173 0.0174 0.0188 0.0219 0.0225 0.0226 0.0248 0.0260 0.0284 0.0303 0.0315 0.0318 0.0320 0.0323 0.0327 0.0329 0.0332 0.0343 0.0345 0.0346 0.0346 0.0347 0.0347 0.0352 0.0359 0.0365 0.0366 0.0370 0.0371 0.0376 0.0384 0.0392 0.0396 0.0419 0.0421 0.0443 0.0445 0.0462 0.0492 0.0506 0.0525 0.0536 0.0539 0.0540 0.0568 0.0569 0.0592 0.0600 0.0603 0.0606 0.0617 0.0619 0.0621 0.0629 0.0632 0.0657 0.0665 0.0675 0.0678 0.0683 0.0684 0.0691 0.0693 0.0707 0.0708 0.0719 0.0722 0.0727 0.0771 0.0773 0.0773 0.0774 0.0791 0.0832 0.0837 0.0845 0.0865 0.0897 0.0907 0.0910 0.0946 0.0961 0.0972 0.1010 0.1056 0.1123 0.1149 0.2153.

**Figure 3:**
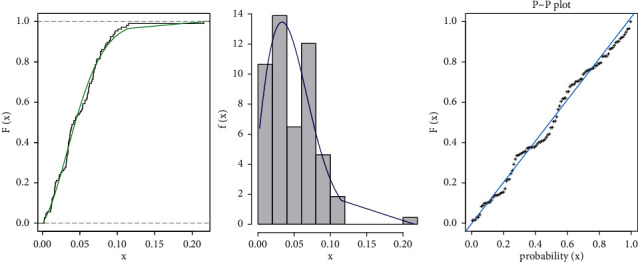
TGL model pdf and CDF estimates, as well as a PP-plot using COVID-19 data of France. Data set (2): COVID-19 of the United Kingdom.

In Table 6, the TGL distribution is fitted to COVID-19 of France country. The TGL model is compared with other competitive models as Mead and Afify [16] proposed the Burr-XII model (KEBXII) with Kumaraswamy exponentiated, Weibull-Lomax (WL) distribution, Odds Exponential-Pareto IV (OEPIV) distribution proposed by Baharith et al. [17], Marshall–Olkin Alpha power Weibull (MOAPW) by Almetwally et al. [18], Marshall–Olkin Alpha power extended Weibull (MOAPEW) by Almetwally [19], Marshall–Olkin alpha power inverse Weibull (MOAPIW) by Basheer et al. [20], Marshall–Olkin alpha power Lomax (MOAPL) by Almongy et al. [21], and Gompertz Lomax (GOLOM) distribution by Oguntunde et al. [11]. According to this result, we note that the estimate of TGL has the best measure where it has the smallest value of Cramer-von Mises (*W*^*∗*^), Anderson-Darling (*A*^*∗*^), and Kolmogorov- Smirnov (KS) statistic along with its *P* value. The fitted TGL, pdf, CDF, and PP-plot of the data set are displayed in Figure 3.

The COVID-19 data in question is from the United Kingdom and spans 82 days, from May 1 to July 16, 2021. This data is formed by using daily ND, daily CC, and daily CD as follows:

0.0023, 0.0023, 0.0023, 0.0046, 0.0065, 0.0067, 0.0069, 0.0069, 0.0091, 0.0093, 0.0093, 0.0093, 0.0111, 0.0115, 0.0116, 0.0116, 0.0119, 0.0133, 0.0136, 0.0138, 0.0138, 0.0159, 0.0161, 0.0162, 0.0162, 0.0162, 0.0163, 0.0180, 0.0187, 0.0202, 0.0207, 0.0208, 0.0225, 0.0230, 0.0230, 0.0239, 0.0245, 0.0251, 0.0255, 0.0255, 0.0271, 0.0275, 0.0295, 0.0297, 0.0300, 0.0302, 0.0312, 0.0314, 0.0326, 0.0346, 0.0349, 0.0350, 0.0355, 0.0379, 0.0384, 0.0394, 0.0394, 0.0412, 0.0419, 0.0425, 0.0461, 0.0464, 0.0468, 0.0471, 0.0495, 0.0501, 0.0521, 0.0571, 0.0588, 0.0597, 0.0628, 0.0679, 0.0685, 0.0715, 0.0766, 0.0780, 0.0942, 0.0960, 0.0988, 0.1223, 0.1343, and 0.1781.

**Figure 4:**
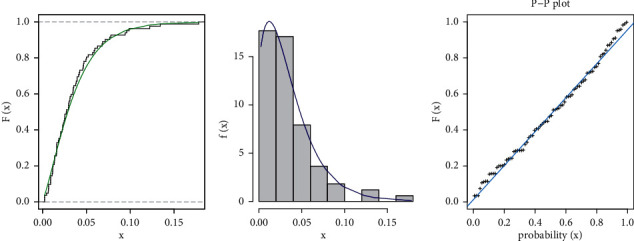
TGL model pdf and CDF estimates, as well as a PP-plot using COVID-19 data of the United Kingdom.

In Table 7, the TGL distribution is fitted to COVID-19 of The United Kingdom country. The TGL model is compared with other competitive models as, KEBXII, WL, OEPIV, MOAPW, MOAPEW, and GOLOM distributions. According to this result, we note that the estimate of TGL has the best measure where it has the smallest value of *W*^*∗*^, *A*^*∗*^, and KS statistic along with its *P* value. The fitted TGL, pdf, CDF, and PP-plot of the data set are displayed in Figure 4.

## 9. Conclusion

We investigate the so-called five-parameter transmuted generalized Lomax distribution in this study. Lomax and Gompertz Lomax (GoLom) distributions are included in the TGL model. The TGL distribution's structural properties are deduced. The maximum likelihood approach is used to estimate the population parameters based on complete and Type-II censored samples. We discussed the Monte Carlo simulation study to estimate point and interval estimation of parameters of TGL distribution based on complete sample sizes and Type-II censored samples. The proposed distribution was applied to two COVID-19 real-world data sets from France and United Kingdom. We compared a new transmuted generalization of the Lomax distribution (TGL) with KEBXII, WL, OEPIV, MOAPW, MOAPEW, and GOLOM distributions. It was shown to provide a better fit than several other models. We hope that the presented model will be used in a variety of fields, including engineering, survival and lifetime data, meteorology, biology, hydrology, economics (income disparity), and others.

## Figures and Tables

**Table 1:** The TGL distribution's special submodels.

No.	Distributions	*γ*	*ϑ*	*δ*	*β*	*π*	Author
1	LOM	*γ*	1	*δ*	0	1	Lomax [15]
2	GOLOM	*γ*	*ϑ*	*δ*	0	*π*	Oguntunde et al. [11]

**Table 2:** Various measures for parameters of TGL distribution based on different schemes of samples: Case 1.

*N*	*M*		Bias	MSE	L.ACI	L.BPCI	L.BTCI	CP (%)
30	30	*π*	0.0046	0.0499	0.8572	0.0992	0.0979	96.40
		*β*	0.0190	0.0392	0.7731	0.0815	0.0812	94.60
		*δ*	0.0241	0.0211	0.5615	0.0551	0.0547	97.80
		*γ*	0.3174	0.5989	2.7682	0.2530	0.2498	95.80
		*ϑ*	−0.0138	0.0392	0.7619	0.0830	0.0834	96.20
	25	*π*	0.0050	0.1381	1.4572	0.1465	0.1461	95.80
		*β*	−0.0752	0.0515	0.8396	0.0841	0.0843	96.30
		*δ*	0.0777	0.0347	0.6641	0.0620	0.0619	96.90
		*γ*	0.3370	0.7575	3.0897	0.2847	0.2880	95.50
		*ϑ*	-0.0238	0.0565	0.9277	0.0900	0.0910	96.70
	20	*π*	0.0445	0.2580	1.9846	0.1882	0.1852	93.20
		*β*	−0.1213	0.0713	0.9333	0.0970	0.0979	98.00
		*δ*	0.1470	0.0769	0.9220	0.0974	0.0974	94.80
		*γ*	0.3673	0.8658	3.3531	0.3672	0.3640	95.00
		*ϑ*	-0.0395	0.0624	0.9670	0.0919	0.0919	96.70

50	50	*π*	0.0139	0.0354	0.7220	0.0689	0.0678	96.70
		*β*	0.0250	0.0372	0.7506	0.0622	0.0636	93.20
		*δ*	0.0146	0.0117	0.4207	0.0251	0.0252	98.10
		*γ*	0.2688	0.4656	2.4598	0.2680	0.2604	95.30
		*ϑ*	−0.0102	0.0286	0.6449	0.0666	0.0656	96.70
	40	*π*	−0.0143	0.1437	1.4834	0.1339	0.1357	94.10
		*β*	−0.0714	0.0482	0.8141	0.0788	0.0781	95.10
		*δ*	0.0763	0.0371	0.6941	0.0603	0.0604	95.30
		*γ*	0.2931	0.5376	2.5974	0.2705	0.2643	96.40
		*ϑ*	−0.0198	0.0505	0.8783	0.0739	0.0719	96.40
	30	*π*	−0.0153	0.2732	2.0492	0.2223	0.2199	94.30
		*β*	−0.0980	0.0587	0.8695	0.0867	0.0875	98.20
		*δ*	0.1424	0.0679	0.8562	0.0953	0.0960	95.10
		*γ*	0.3750	0.5968	2.8313	0.2948	0.3059	95.60
		*ϑ*	−0.0347	0.0548	0.9084	0.0801	0.0798	97.70

100	100	*π*	0.0031	0.0190	0.5275	0.0444	0.0447	96.60
		*β*	0.0089	0.0305	0.5867	0.0581	0.0578	93.20
		*δ*	0.0054	0.0023	0.1851	0.0163	0.0162	95.60
		*γ*	0.1932	0.2261	1.7040	0.1498	0.1481	95.00
		*ϑ*	−0.0103	0.0149	0.4653	0.0476	0.0480	97.00
	90	*π*	0.0044	0.0490	0.8679	0.0984	0.0982	95.20
		*β*	−0.0284	0.0312	0.6836	0.0647	0.0645	93.80
		*δ*	0.0275	0.0052	0.2603	0.0242	0.0243	95.00
		*γ*	0.2084	0.3174	2.0528	0.1766	0.1782	95.10
		*ϑ*	−0.0127	0.0227	0.5874	0.0512	0.0524	96.30
	70	*π*	−0.0750	0.1673	1.5771	0.1576	0.1573	94.60
		*β*	−0.0488	0.0409	0.7697	0.0850	0.0846	98.70
		*δ*	0.0869	0.0277	0.5562	0.0572	0.0562	94.90
		*γ*	0.2216	0.4024	2.3311	0.2574	0.2514	96.10
		*ϑ*	−0.0177	0.0366	0.7499	0.0684	0.0681	97.70

**Table 3:** Various measures for parameters of TGL distribution based on different schemes of samples: Case 2.

N	m		Bias	MSE	L.ACI	L.BPCI	L.BTCI	CP (%)
30	30	*π*	0.0109	0.1883	1.6472	0.1585	0.1567	94.60
		*β*	−0.3184	2.3256	4.2623	0.3338	0.3204	99.10
		*δ*	0.1568	0.1692	1.4916	0.1371	0.1378	95.20
		*γ*	0.1809	0.6919	3.0693	0.2352	0.2353	97.10
		*ϑ*	−0.0090	0.0501	0.8773	0.0941	0.0941	94.60
	25	*π*	0.0316	0.5118	2.8058	0.2225	0.2250	92.90
		*β*	−0.4821	4.0856	7.6986	0.4347	0.4033	98.00
		*δ*	0.2003	0.2467	1.7824	0.1643	0.1644	95.90
		*γ*	0.2844	0.7288	3.1515	0.2746	0.2742	95.00
		*ϑ*	0.0272	0.0839	1.1311	0.1115	0.1128	94.30
	20	*π*	0.0657	0.6546	3.1725	0.3272	0.3284	93.50
		*β*	−0.8510	13.7077	14.1318	1.1032	1.0926	96.90
		*δ*	0.2369	0.3087	1.9713	0.1659	0.1649	96.70
		*γ*	0.3023	0.7742	3.2409	0.2864	0.2825	93.70
		*ϑ*	0.0342	0.1215	1.3639	0.1106	0.1107	94.00

50	50	*π*	0.0108	0.0817	1.0769	0.1063	0.1059	93.07
		*β*	−0.0870	0.0945	1.1618	0.1227	0.1224	93.07
		*δ*	0.0485	0.0603	0.9486	0.0939	0.0922	97.03
		*γ*	0.1485	0.2533	1.8946	0.1939	0.1925	96.04
		*ϑ*	−0.0040	0.0389	0.7607	0.0754	0.0768	94.06
	40	*π*	0.0183	0.3512	2.3230	0.2385	0.2367	95.00
		*β*	−0.2663	1.0665	3.9132	0.2870	0.2683	99.20
		*δ*	0.1512	0.1673	1.4905	0.1475	0.1488	95.60
	30	*γ*	0.1849	0.3970	2.3623	0.2572	0.2540	94.80
		*ϑ*	0.0100	0.0540	0.9104	0.0840	0.0817	95.10
		*π*	−0.0344	0.7091	3.2999	0.4086	0.4018	93.60
		*β*	−0.5091	4.3847	7.9660	0.4242	0.4161	98.30
		*δ*	0.1930	0.2781	1.9247	0.1890	0.1890	96.70
		*γ*	0.3312	0.6945	2.9991	0.2559	0.2592	94.30
		*ϑ*	0.0706	0.0991	1.2345	0.1139	0.1134	94.10

100	100	*π*	0.0094	0.0397	0.7641	0.0725	0.0726	93.53
		*β*	−0.0081	0.0623	0.9812	0.0983	0.0986	95.88
		*δ*	0.0309	0.0348	0.7228	0.0663	0.0664	95.88
		*γ*	0.0541	0.1002	1.2261	0.1169	0.1169	94.71
		*ϑ*	−0.0106	0.0082	0.3495	0.0338	0.0340	94.71
	90	*π*	−0.0102	0.0911	1.1829	0.1255	0.1258	95.20
		*β*	−0.0726	0.0746	1.0323	0.1064	0.1061	98.10
		*δ*	0.0405	0.0500	0.8625	0.0962	0.0975	95.00
		*γ*	0.0876	0.1327	1.3866	0.1314	0.1314	95.40
		*ϑ*	0.0136	0.0158	0.4898	0.0517	0.0507	94.60
	70	*π*	−0.0106	0.2094	1.7942	0.1858	0.1806	95.10
		*β*	−0.1500	0.1102	1.1617	0.1116	0.1120	96.50
		*δ*	0.0757	0.1098	1.2650	0.1282	0.1294	96.00
		*γ*	0.1398	0.1782	1.5624	0.1573	0.1573	94.50
		*ϑ*	0.0151	0.0230	0.5924	0.0522	0.0522	96.00

**Table 4:** Various measures for parameters of TGL distribution based on different schemes of samples: Case 3.

N	M		Bias	MSE	L.ACI	L.BPCI	L.BTCI	CP (%)
30	30	*π*	0.0937	0.1825	1.6349	0.1569	0.1576	99.89
		*β*	−0.9293	1.0697	4.0882	1.2977	1.3166	93.69
		*δ*	0.0507	0.0593	0.9339	0.0523	0.0519	97.01
		*γ*	0.7467	2.3515	5.2533	0.7690	0.7595	95.90
		*ϑ*	−0.0886	0.1389	1.4201	0.1467	0.1464	98.45
	25	*π*	0.0675	0.2530	1.9547	0.2090	0.2062	98.60
		*β*	−1.0851	2.8773	4.3929	2.0501	1.9617	95.10
		*δ*	0.0922	0.0619	0.9060	0.0849	0.0850	94.80
		*γ*	0.8103	2.5817	5.4576	0.4411	0.4389	96.50
		*ϑ*	−0.0643	0.1778	1.6342	0.1544	0.1553	97.20
	20	*π*	0.0563	0.3745	2.3900	0.2332	0.2358	98.50
		*β*	−1.4870	3.2350	5.2192	1.9231	1.9180	94.50
		*δ*	0.1298	0.0896	1.0575	0.1038	0.1037	95.30
		*γ*	0.9029	2.7732	5.4879	0.5950	0.5936	95.20
		*ϑ*	−0.0555	0.2429	1.9205	0.1908	0.1920	97.00

50	50	*π*	0.0748	0.1549	1.5154	0.1457	0.1476	99.60
		*β*	−0.3842	0.9235	3.6210	0.5458	0.5468	94.40
		*δ*	0.0510	0.0486	0.8415	0.0648	0.0651	96.30
		*γ*	0.4395	0.9975	3.5173	0.3065	0.2983	94.70
		*ϑ*	−0.0925	0.1053	1.2198	0.1239	0.1228	98.50
	40	*π*	0.0557	0.2313	1.8735	0.1751	0.1735	98.20
		*β*	−0.9798	1.1929	4.0658	1.3002	1.2948	94.80
		*δ*	0.0855	0.0769	1.0348	0.0723	0.0728	95.70
		*γ*	0.6323	1.8210	4.6755	0.3789	0.3788	95.60
		*ϑ*	−0.1014	0.1360	1.3908	0.1445	0.1457	98.10
	30	*π*	0.0307	0.3267	2.2384	0.2249	0.2275	98.60
		*β*	−1.0355	3.2609	4.9372	2.1615	2.1135	94.00
		*δ*	0.1278	0.0792	0.9260	0.0974	0.0974	95.50
		*γ*	0.6536	1.9676	4.8675	0.5197	0.5227	94.30
		*ϑ*	−0.0765	0.1560	1.5196	0.1469	0.1522	97.00

100	100	*π*	0.0770	0.0972	1.1846	0.1112	0.1113	97.70
		*β*	−0.0124	0.5159	2.8165	0.3334	0.3336	98.50
		*δ*	0.0237	0.0152	0.4742	0.0470	0.0468	94.30
		*γ*	0.2246	0.4043	2.3330	0.1685	0.1660	97.40
		*ϑ*	−0.0733	0.0549	0.8724	0.0847	0.0855	96.10
	90	*π*	0.0788	0.1203	1.3248	0.1411	0.1409	97.00
		*β*	−0.0530	0.5808	2.9817	0.2731	0.2639	98.20
		*δ*	0.0311	0.0179	0.5107	0.0523	0.0512	94.90
		*γ*	0.2587	0.5089	2.4834	0.2217	0.2187	97.50
		*ϑ*	−0.0816	0.0613	0.9170	0.0852	0.0854	96.30
	70	*π*	0.0427	0.1847	1.6773	0.1740	0.1747	96.70
		*β*	−0.1007	0.6838	3.2191	0.3718	0.3522	97.70
		*δ*	0.0656	0.0326	0.6602	0.0657	0.0654	93.80
		*γ*	0.2924	0.5365	2.6340	0.2452	0.2441	96.60
		*ϑ*	−0.0840	0.0855	1.0983	0.0993	0.0991	98.30

**Table 5:** Various measures for parameters of TGL distribution based on different schemes of samples: Case 4.

N	M		Bias	MSE	L.ACI	L.BPCI	L.BTCI	CP (%)
30	30	*π*	0.0457	0.8716	3.6572	0.3452	0.3467	93.10
		*β*	−0.7394	2.9165	3.1121	1.0574	1.0499	95.00
		*δ*	0.2040	0.1219	1.1113	0.1042	0.1047	93.80
		*γ*	0.0972	1.1947	4.2698	0.3181	0.3179	94.70
		*ϑ*	−0.0600	0.5720	2.9403	0.2416	0.2414	94.00
	25	*π*	−0.0546	1.4718	4.7532	0.4506	0.4508	91.50
		*β*	−0.8497	3.0780	3.2331	1.1783	1.1561	93.00
		*δ*	0.2697	0.3005	1.8717	0.1570	0.1558	96.80
		*γ*	0.2684	1.5735	4.8057	0.4081	0.4142	92.60
		*ϑ*	−0.0657	0.9053	3.7228	0.3539	0.3501	95.40
	20	*π*	−0.1183	1.8618	5.3313	0.5021	0.5052	95.60
		*β*	−0.9115	3.5046	3.9777	1.1938	1.1806	93.60
		*δ*	0.3144	0.3758	2.0640	0.2992	0.2867	96.30
		*γ*	0.3536	2.0095	5.3839	0.5360	0.5328	93.50
		*ϑ*	−0.0967	1.1029	4.1143	0.3862	0.3746	96.30

50	50	*π*	0.0714	0.5478	2.8490	0.3028	0.3013	96.90
		*β*	−0.1603	1.8194	2.5870	0.6000	0.5338	98.00
		*δ*	0.1516	0.0740	0.8855	0.0822	0.0830	94.00
		*γ*	−0.0048	0.3142	2.2536	0.1978	0.1976	96.90
		*ϑ*	−0.1249	0.3481	2.2389	0.2402	0.2411	97.40
	40	*π*	0.0800	0.7323	3.3415	0.3160	0.3159	96.10
		*β*	−0.3022	2.0353	2.9569	1.0054	0.8703	97.70
		*δ*	0.1595	0.0794	0.9111	0.0966	0.0970	95.20
		*γ*	−0.0053	0.3584	2.3478	0.2355	0.2312	96.30
		*ϑ*	−0.1345	0.4580	2.6940	0.2957	0.2946	97.20
	30	*π*	0.1254	0.9918	3.9046	0.3661	0.3689	95.10
		*β*	−0.6051	2.1776	3.0807	1.1412	1.0517	96.70
		*δ*	0.1817	0.2014	1.6094	0.1155	0.1167	97.50
		*γ*	0.0084	0.5461	2.8893	0.2832	0.2732	96.00
		*ϑ*	−0.1449	0.5641	2.8903	0.3036	0.3074	96.00

100	100	*π*	0.0796	0.2255	1.8359	0.1756	0.1770	97.10
		*β*	0.0607	0.1229	1.3542	0.1123	0.1117	98.30
		*δ*	0.0620	0.0165	0.4407	0.0405	0.0401	95.20
		*γ*	−0.0551	0.0639	0.9675	0.1007	0.1005	94.90
		*ϑ*	−0.1031	0.1754	1.5918	0.1555	0.1543	96.00
	90	*π*	0.0965	0.3391	2.2524	0.2311	0.2307	96.70
		*β*	0.0668	0.1273	1.3743	0.1092	0.1095	99.30
		*δ*	0.0788	0.0233	0.5124	0.0461	0.0462	95.30
		*γ*	−0.0468	0.0696	1.0182	0.0878	0.0872	96.40
		*ϑ*	−0.1320	0.2347	1.8281	0.1765	0.1761	98.10
	70	*π*	0.0949	0.4762	2.6806	0.3019	0.3017	97.00
		*β*	0.0006	1.3674	4.5861	0.4831	0.4145	99.30
		*δ*	0.0968	0.0389	0.6739	0.0468	0.0470	96.60
		*γ*	−0.0358	0.1282	1.3972	0.1466	0.1461	96.90
		*ϑ*	−0.1416	0.2931	2.0494	0.2083	0.2070	98.70

**Table 6:** The TGL model's and other competing models' analytical results using COVID-19 data of France.

		*π*	*β*	*δ*	*γ*	*ϑ*	KS	*P* value	*W* ^*∗*^	*A* ^*∗*^
TGL	Estimates	8.3588	0.9078	0.2296	106.5058	0.1131	**0.0660**	**0.7348**	**0.0795**	**0.4964**
	SE	3.3853	0.1102	2.2730	1.7397	1.3473				

KEBXII	Estimates	1.5895	0.7072	52.8189	1.4050	1.7894	0.0719	0.6327	0.1013	0.6654
	SE	1.1360	0.3115	6.6434	0.2670	1.0125				

WL	Estimates	2.8707	1.5466	1.0213	0.1104	—	0.0711	0.6466	0.0968	0.6432
	SE	20.4928	0.3118	0.9636	0.4204					

OEPIV	Estimates	3.7914	0.6310	0.8663	0.1134	—	0.0721	0.6281	0.1024	0.6726
	SE	22.6007	0.1039	1.2677	0.3183					

MOAPIW	Estimates	3171.2439	0.6122	0.0026	0.1791	—	0.1100	0.1462	0.4416	2.7501
	SE	1410.3818	0.0414	0.0004	0.0317					

MOAPW	Estimates	7.7493	1.0168	2.1276	0.0244	—	0.0663	0.7292	0.0909	0.5547
	SE	26.4113	0.3237	2.7080	0.0155					

MOAPEW	Estimates	3.2759	0.8594	6.2503	18.8847	23.6501	0.0673	0.7118	0.0944	0.5600
	SE	6.4510	0.3284	7.5313	1.5417	36.6454				

MOAPL	Estimates	1.0858	29.2003	7.7492	0.5695		0.0667	0.7223	0.0941	0.5544
	SE	4.9184	55.2298	17.9049	1.1921					

GOLOM	Estimates	0.6174	—	6.5887	5.1836	0.3340	0.0912	0.3299	0.0798	0.5392
	SE	0.3285		3.6730	2.7694	0.2097				

**Table 7:** The TGL model's and other competing models' analytical results using COVID-19 data of The United Kingdom.

		*π*	*β*	*δ*	*γ*	*ϑ*	KS	*P* value	*W* ^*∗*^	*A* ^*∗*^
TGL	Estimates	0.3759	0.7269	3.7995	197.6717	0.0110	**0.0579**	**0.9313**	**0.0520**	**0.3666**
	SE	0.1098	0.2824	1.0761	81.6337	0.0052				

KEBXII	Estimates	9.2969	0.0223	45.1190	5.8785	1.7442	0.0686	0.8093	0.0574	0.4058
	SE	4.9136	0.0133	13.0012	0.0024	0.0329				

WL	Estimates	209.1293	1.2386	87.0044	253.2098	—	0.0589	0.9221	0.0573	0.4038
	SE	427.5093	0.0999	267.0487	985.0169	—				

OEPIV	Estimates	344.2589	0.7995	4.5559	13.9408	—	0.0600	0.9120	0.0557	0.3935
	SE	89.5478	0.0553	2.5642	1.9995	—				

MOAPW	Estimates	8.3728	0.6544	4.8152	0.0067	—	0.0620	0.8912	0.0614	0.4256
	SE	11.2011	0.0629	3.2705	0.0020	—				

MOAPEW	Estimates	12.7782	0.6446	3.6328	7.3977	11.8345	0.0643	0.8646	0.0562	0.3930
	SE	14.6837	0.2803	7.6985	11.0566	12.0845				

GOLOM	Estimates	1.4935	-	1.4072	5.3373	3.1370	0.1003	0.3574	0.0719	0.4967
	SE	7.5715		8.0245	17.3575	13.6175				

## Data Availability

All data used to support the findings of the study are available within the article.
